# Thickness-controlled electronic structure and thermoelectric performance of ultrathin SnS2 nanosheets

**DOI:** 10.1038/s41598-017-09572-9

**Published:** 2017-08-21

**Authors:** Jun Li, Jinni Shen, Zuju Ma, Kechen Wu

**Affiliations:** 1 State Key Laboratory of Structural Chemistry, Fujian Institute of Research on the Structure of Matter, Chinese Academy of Sciences, Fuzhou, Fujian, 350002 People’s Republic of China; 2 University of Chinese Academy of Sciences, Beijing, 100049 People’s Republic of China; 3 College of Materials Science and Engineering, Fuzhou University, Fuzhou, 350108 People’s Republic of China

## Abstract

The thermoelectric conversion efficiency of a material relies on a dimensionless parameter (*ZT* = *S* ^2^ *σT*/*κ*). It is a great challenge in enhancing the *ZT* value basically due to that the related transport factors of most of the bulk materials are inter-conditioned to each other, making it very difficult to simultaneously optimize these parameters. In this report, the negative correlation between power factor and thermal conductivity of nano-scaled SnS_2_ multilayers is predicted by high-level first-principle computations combined with Boltzmann transport theory. By diminishing the thickness of SnS_2_ nanosheet to about 3 L, the *S* and *σ* along *a* direction simultaneously increase whereas *κ* decreases, achieving a high *ZT* value of 1.87 at 800 K. The microscopic mechanisms for this unusual negative correlation in nano-scaled two dimensional (2D) material are elucidated and attributed to the quantum confinement effect. The results may open a way to explore the high *ZT* thermoelectric nano-devices for the practical thermoelectric applications.

## Introduction

Thermoelectric materials have attracted much attention due to their meaningful applications in directly converting heat to electricity, which are expected as promising future power generation^1–3^. The performance of a thermoelectric material is quantified by the dimensionless figure of merit *ZT* = *S* ^2^ *σT*/*κ*, where *S* is the Seebeck coefficient, *σ* is the electrical conductivity, *T* is the absolute temperature, and *κ* is the total thermal conductivity, consisting of lattice (*κ*_*ph*_) and electronic (*κ*_*el*_) contributions. The quantity *S* ^2^ *σ* is called the power factor (*PF*), which characterizes the capability of electrical power under a given temperature difference. Realizing a good thermoelectric material should have a large *ZT* value, which requires high *PF* and low *κ*. However, it is a great challenge to achieve a high *ZT* value since these transport parameters are interlinked, optimizing one coefficient often leads to another being adversely affected. To enhance the *ZT* value, the band structure of materials is always modified, due to the electron functions of *S*, *σ*, and *κ*_*el*_.

Nanostructuring has been suggested to be an effective strategy to improve the performance of thermoelectric materials. Many reported nano-materials obtained higher *ZT* values than their bulk counterparts due to the decreased *κ* ^4–7^. The grain boundaries and interfaces within the nanostructures could facilitate the phonon scattering, contributing to the reduction in lattice thermal conductivity^8–11^. However, most increase in *ZT* by nanostructuring has been deem that the thermal conductivity considerably reduced but without significantly affecting the power factor. In fact, the negative correlation of the two factors, for example, the reduced *κ* and increased *PF* is indeed the ideal strategy to improve the *ZT* value. Intriguingly, Hicks and Dresselhaus proposed that some low-dimensional or nano-structured thermoelectric materials could exhibit much higher *ZT* values on account of the improved *PF* induced by the quantum confinement effect^12, 13^. Such enhancement can be just ascribed to the increased *S* caused by the high density of states near the Fermi level. The opposite correlation and coupling among the *S* and *σ* make it a challenging task to simultaneous optimize this two parameters to achieve high *PF*. Thus, it is a great deal of meaningful to devise strategies to simultaneously increase *S* and *σ* by tuning the charge carrier concentrations or the electronic band structure via low-dimensional solutions. Relative researches are rarely reported except the isovalent sulfur doped Bi_2_Te_2_Se nanoplates, the doping-induced multifold increase in the effective density of states contributes to simultaneous increase of *S* and *σ* ^14^. The results successfully break the trade-off between parameters for nanostructured compounds, which will be a new insight into designing two dimensional (2D) high-performanced thermoelectric materials.

SnS_2_^15–17^, a typical metal dichalcogenide with structurally analogous to many layered excellent thermoelectric performance materials, such as SnSe^18, 19^, and phosphorous^20, 21^. The large interlayer van der Walls spacings and weak interlayer interactions of these layered compound make them easily be exfoliated into flakes. Some unexpected properties always be discovered when the layer semiconductors comes from bulk to 2D materials^22–24^. Furthermore, these atomic-scale thickness flakes turn out to exhibit excellent thermoelectric performance, due to the high carrier mobility and low thermal conductivity^22, 25–29^. Moreover, the electrical conductivity of SnS_2_ films can be improved as the thickness of samples decreased^30^. These works all suggest that it is worth to conduct a systematic study on the thermoelectric performance of 2D SnS_2_ nanosheets.

Motived by inspiring unusual electrical and thermal transport phenomenon from bulk to nanosheet of SnS_2_, we focus on investing the thickness effect on the electronic and thermoelectric properties of 2D SnS_2_ multilayers. The results reveal that electrical conductivities and Seebeck coefficient of multilayer SnS_2_ nanosheets exhibits unusual simultaneous increasing when the thickness approaching the exciton Bohr radius of bulk SnS_2_. Moreover, the *κ* decreased as expected with the diminishing of the thickness of SnS_2_ nanosheets, owing to the increased phonon scattering. Thus, the increased power factor and degenerated *κ* accounts for the ideal negative correlation to the decreased thickness of SnS_2_ nanosheets, resulting in the improvement of *ZT* value. At 800 K, the peak *ZT* value of 3-layers (3 L) SnS_2_ sheets reaches 1.87, enhanced nearly 3 times compared to that of the bulk phase at the same temperature with optimal doping carrier concentration.

## Results and Discussion

### Geometric structure of SnS_2_ nanosheets

**Figure 1:**
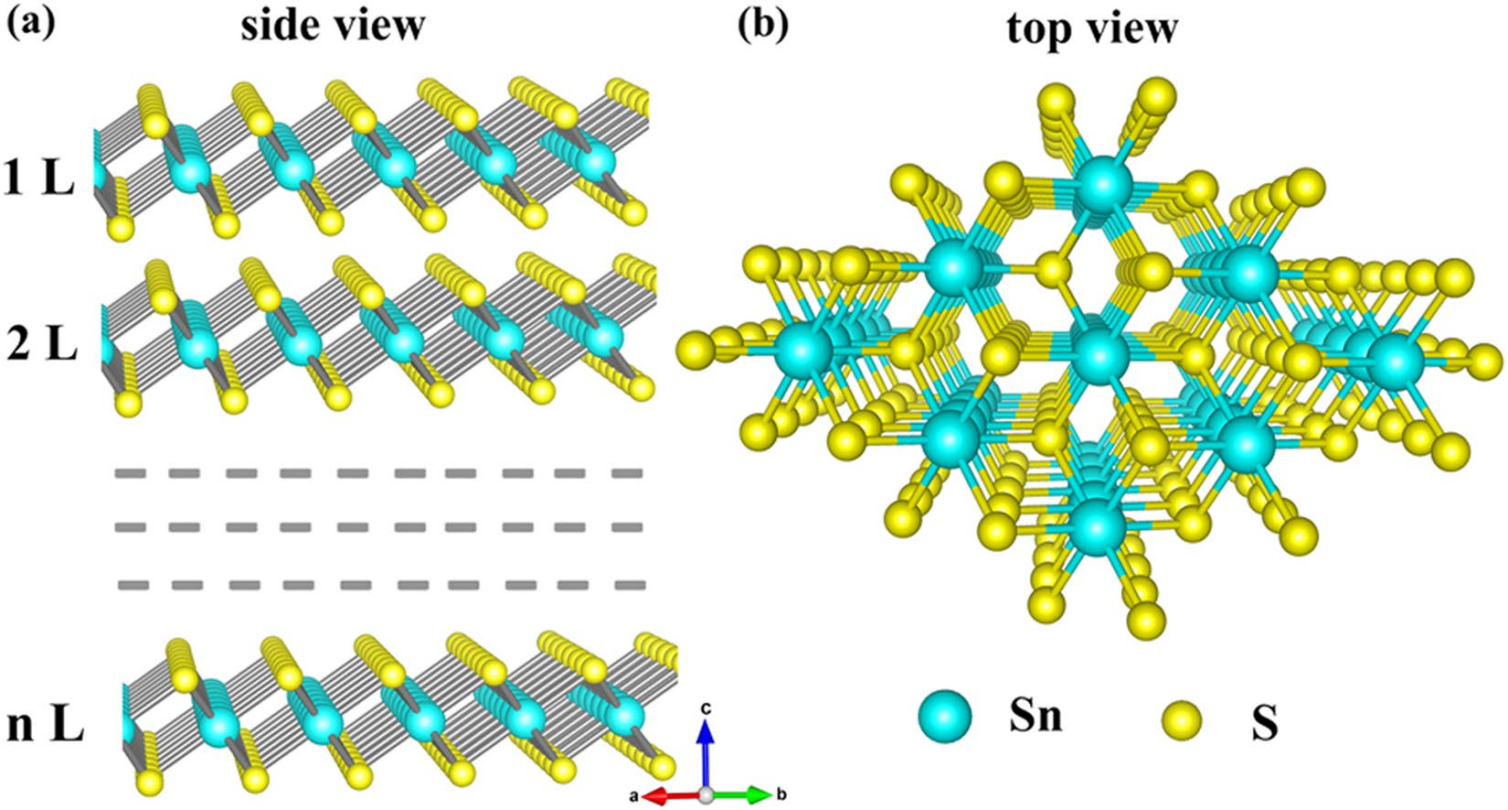
Schematic structures of SnS_2_ nanosheets. (**a**) Side view of the multilayers, (**b**) Top view.

With a benchmark test for the calculation method and parameterization used in this work, we systematically investigate the structural, electronic and transport properties of the SnS_2_ nanosheets with different thickness. The optimized 2D SnS_2_ sheets adopts the CdI_2_ layered structure with hexagonal lattice, in which tin atoms are located in the octahedral sites between two hexagonally close packed sulfur slabs to form a sandwich structure. The SnS_2_ layer is stacked on top of one other along the crystallographic *c*-axis and is held together by weak Van der Waals forces, as shown in Fig. 1(a). The separation between contiguous layers is around 0.59 nm, which is in highly agreement with that of the recent synthesized SnS_2_ nanosheets (0.60 nm)^31^. The layered structure with the weak interlayer force makes it to be easily cleaved perpendicular to the *c*-axis producing atomically smooth surfaces. The geometry structure for SnS_2_ nanosheets different thicknesses are optimized by PBE-D2 method with van der Waals interaction in consideration^32^. The lattice constants for various layers samples are similar, possessing the value of *a* = 3.643 Å, which is in reasonable agreement the previous theoretical and experimental results^33, 34^. A detailed analysis on the sample thickness dependence of geometric parameters of SnS_2_ nanosheets, such as the bond length, bond angle and inter-layer distance can be found in Fig. S1 and Table S1 of Supplementary Information.

### Electronic structure of multilayer SnS_2_ nanosheets

**Figure 2:**
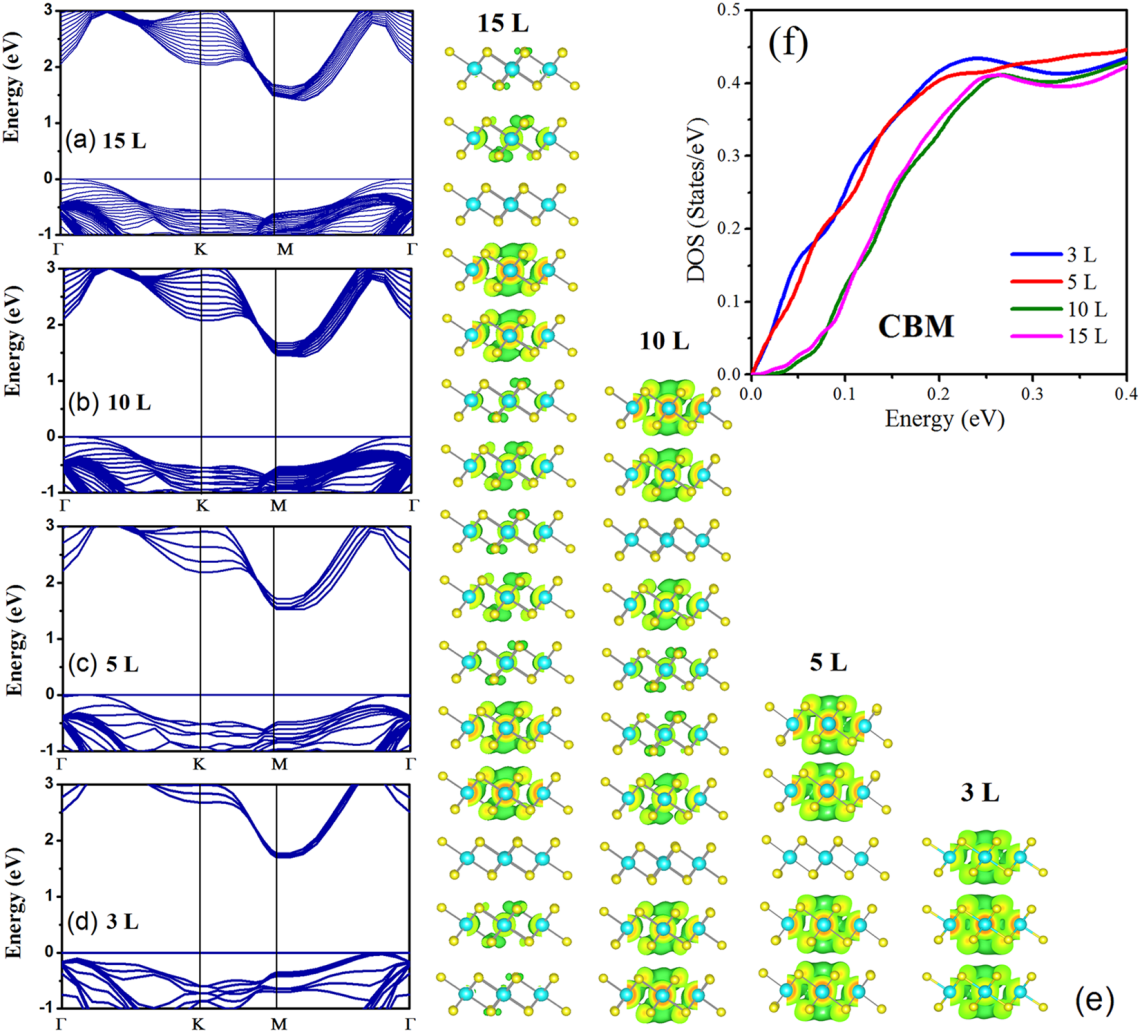
Electronic band structure of SnS_2_ nanosheets in different layers: 15 L (**a**), 10 L (**b**), 5 L (**c**) and 3 L (**d**). (**e**) Electron density distribution of conduction band minimum for SnS_2_ nanosheets with different thickness. (**f**) The density of states (DOS) of conduction band minimum in per layer of SnS_2_ nanosheets in 15 L, 10 L, 5 L and 3 L.

To explore the origin of thickness dependence of thermoelectric transport properties, we calculate the electronic band structure of SnS_2_ nanosheets with different layers, which is closely related to the Seebeck coefficient and electrical conductivity. Figure 2(a–d) present the electronic band structure of SnS_2_ nanosheets in 15 L, 10 L, 5 L and 3 L, respectively. It can be observed that the conduction band edge of SnS_2_ splits into different subsets corresponding to the number of sheet layers. For the different layers SnS_2_ nanosheets, the conduction band minimum (CBM) lies at M point and the valance band maximum (VBM) is located on the Γ–M path, which indicates that the reduction of dimension maintains the indirect band structure of SnS_2_. Furthermore, the SnS_2_ sheets samples in various thicknesses show the similar band structure characteristic near the CBM, signifying the similar electron effective mass. The calculated and experimental band gap for 2D SnS_2_ sheets corresponding to different layers and bulk SnS_2_ are collected and visualized in Fig. S2 of Supplementary Information. With the thickness decreased, the weakened interaction between the layers contributes to smaller band dispersion and larger band gap due to the quantum confinement effect. The enlarged band gap is favor to overcome the high temperature bipolar conduction problem which may degrade the thermoelectric performance.

When the number of layers sets at 5, the thickness of SnS_2_ nanosheets is about 3 nm, which is quite close to the exciton Bohr radius (*a*_*ex*_) of bulk SnS_2_. The exciton Bohr radius for SnS_2_ can be estimated to be around 3.64 nm using the below equation and values of dielectric constants and effective mass from previous report^35^:

1 $${a}_{ex}=\,{a}_{H}{\varepsilon }_{r}\frac{{m}_{0}}{\nu }\,$$

2 $$\nu =\,{m}_{e}^{\ast }{m}_{h}^{\ast }/({m}_{e}^{\ast }+{m}_{h}^{\ast })$$

where *a*_*H*_ = 0.53 Å is Bohr radius of hydrogen atom, and *m*_0_, $\nu$, ${m}_{e}^{\ast }$ = 0.43 *m*_0_, ${m}_{h}^{\ast }$ = 0.61 *m*_0_, as calculated in our previous report^15^, are the mass of a hydrogen atom, reduced mass, and the electron effective masses of the longitudinal contribution (perpendicular to *a-b* plane), electron effective masses of the horizontal contribution (parallel to *a-b* plane) respectively. Besides, *ε*_*r*_ = 17.7 is the high frequency dielectric constant for bulk SnS_2_^36^. Generally, with the dimension decreasing from bulk to 2D scale, size effect generally becomes pronounced. Herein, we find that the carrier density of SnS_2_ nanosheets with various thicknesses exhibits significant different distribution. As the intri*n*sic *n*-type semiconductor for SnS_2_ in films^30, 37^, electrons distribution of conduction band minimum (CBM) dominates the carrier transport. The calculated band decomposed charge density for CBM of SnS_2_ nanosheets in various layers are shownin Fig. 2(e). For 15 layers (15 L) and 10 layers (10 L) samples, electrons of CBM just distribute on 6 layers among them, and most of layers are in absence of electrons distribution. With the thickness decreasing, 4 layers in 5-layer (5 L) sample and total layers of 3-layer (3 L) sample have the electrons occupation, indicating the denser electrons density inter layer for thinner nanosheets. It can be predicted that the electron transfer perpendicular to the surface of layers would be confined due to the quantum confinement effect with the dimension in this direction close to quantum size. Such a phenomenon induces the accumulation of electron on the surface of in-plane, which is beneficial to the enhancement of electrical conductivity.

The conduction band density of states (DOS) of per layer for SnS_2_ nanosheets with different thicknesses is shown in Fig. 2(f). For the sake of more convenient comparison, the conduction band minimum of different layers is shift to 0 eV. Based on the Boltzmann transport theory, the Seebeck coefficient is expressed in the Mott equation^38^:

3 $$S=\,\frac{{\pi }^{2}}{3}\frac{{k}^{2}T}{e}{\frac{{\rm{d}}\mathrm{ln}\sigma (E)}{{\rm{d}}E}|}_{E={E}_{f}}$$

The electronic conductivity *σ*(*E*) is related to the density of states (DOS) at *E*. Thus the larger slope $(\frac{{\rm{d}}\,\mathrm{ln}\,\sigma (E)}{{\rm{d}}E})$ of DOS is expected to have a higher Seebeck coefficient. It is shown that the DOS of nanosheets in 5 L and 3 L holds larger slope than that of 15 L and 10 L, indicating higher Seebeck coefficient for the former ones. Furthermore, the asymmetry of the DOS near the Fermi energy are pronoued when the layers number of sheets decreased (See Fig. S3 in Supplementary Information), which is in favor to the enhancement of electrical conductivity.

### Thickness dependence of thermoelectric properties

**Figure 3:**
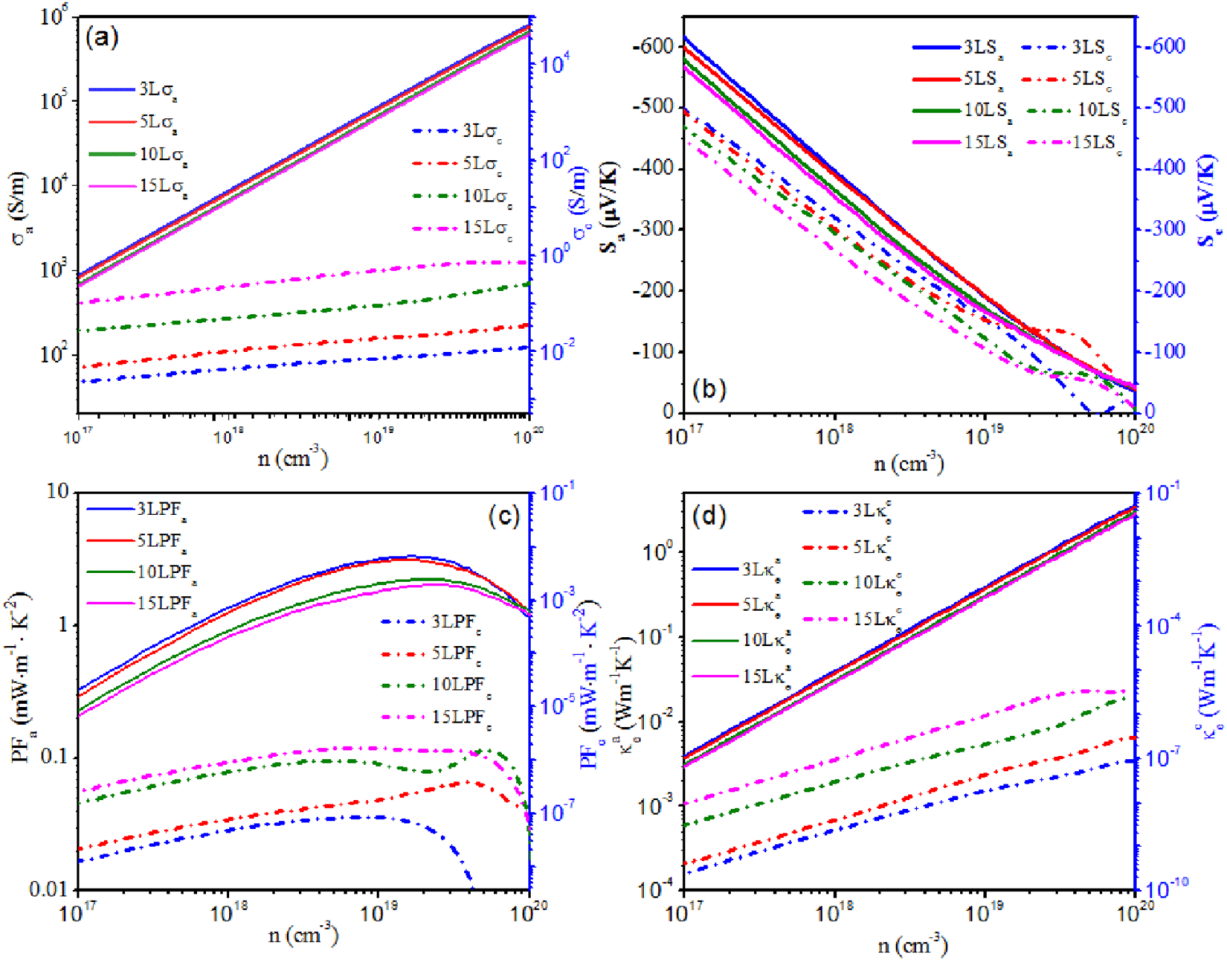
Calculated anisotropic electronic transport properties: electrical conductivity *σ*_*a*_ and *σ*_*c*_ (**a**), Seebeck coefficients *S*_*a*_ and *S*_*c*_ (**b**), power factor *PF*_*a*_ and *PF*_*c*_ (**c**) and electronic thermal conductivity ${\kappa }_{e}^{a}$ and ${\kappa }_{e}^{c}$ (**d**) as a function of carrier concentration for multilayer SnS_2_ nanosheets with 3 L, 5 L, 10 L, 15 L. *a* and *c* refer to the transport properties of SnS_2_ along the *a-b* plane and *c* direction, respectively.

The anisotropy and anharmonicity is a significant issue for layered materials, thus the anisotropic transport properties of SnS_2_ nanosheets are calculated. The electrical transport properties can be obtained using the BoltzTrap code based on the semiclassic Boltzmann transport theory^39^. The directly derived electrical conductivity (*σ*) from this code is expressed as the ratio of *σ* to relaxation time (*τ*). Typically, constant relaxation time approximation is adopted to yield *σ* for simplicity and convenience, which has been shown to provide good description of electrical conductivity in variety of thermoelectric materials^40–42^. Herein, the *τ* value (1.37 × 10^−15^ s) of the bulk SnS_2_ is utilized to acquire the *σ* of the nanosheets due to the similar bonding features between them. The electrical conductivity on the *a-b* plane and in the *c*-axis direction as a function of carrier concentration at temperature *T* = 300 K are shown in Fig. 3(a) for SnS_2_ nanosheets with different layers. In our calculation, a siginificant boost of *σ*_*a*_ can be observed with the increase of electron concentration. However, the sensitivity of *σ*_*c*_ to the carrier concentration is much weaker than that of *σ*_*a*_. Taking the 3 L sample as an example, as carrier concentration increases from 10^17^ to 10^20^ cm^−3^, the *σ* varies rapidly from 9 × 10^2^ Ω^−1^·m^−1^ to 8.3 × 10^5^ Ω^−1^·m^−1^ for *a* direction while from 0.005 to 0.02 Ω^−1^·m^−1^ for *c* direction. At the same carrier concentration, the value of *σ* along the *a-b* plane is much greater than that along the *c* direction, which exhibits strong anisotropy. More important, the anisotropy of electrical conductivity (*σ*_*a*_/*σ*_*c*_) for 2D SnS_2_ sheet is significantly enhanced with the thickness decreasing from 15 L to 3 L. Because the value of *σ*_*a*_ increases with the thickness diminishing, however, the value of *σ*_*c*_ decreases contrarily. The main reason can be attributed to the quantum confinement effect of 2D nanosheets. From the formula of electrical conductivity *σ* = *neμ*, the enhanced *σ* can be ascribed to the increased electron concentration, *n*, or the improved carrier mobility, *μ*. As referred above, the enlarged electrical conductivity of *a-b* plane induced by thickness decreasing might originate from the high electron concentration caused by the accumulated electrons of interlayers. When the number of layers for SnS_2_ sheet decreases from 15 to 3, the thickness of samples decreased from around 9 nm to 1.8 nm. The quantum confinement effect induced by geometric dimensionality is expected. As well known, semiconductor nanoparticles have tunable optical and electronic properties as their size approaches quantum horizon. With the thickness of SnS_2_ nanosheets decreasing, the dimension in the direction of *c*-axis gradually approaches quantum horizon. When the thickness of SnS_2_ nanosheets closes to *a*_*ex*_, the transfer of electrons will be confined in this direction. With the carrier electrons squeezed in such a narrow space, the density of electrons in the *a-b* plane are accumulated gradually. The confinement for electrons to cross the interlayer leads to the preferring in-plane current transport, and further the high electrical conductivity. The anisotropic structure between in-plane and out-plane contribute to the anisotropic transport property and the higher electron transport resistance along the *c*-axis orientation.

The Seebeck coefficients (*S*) of *n*-type SnS_2_ nanosheets with different layers as functions of carrier concentration at *T* = 300 K in *a* and *c* direction are shown in Fig. 3(b). As can be seen, at low and medium electron concentration, the *S* in *a* direction is higher than that of *c* direction, which exhibits smaller anisotropy comparing with the electrical conductivity. The anisotropy is further degraded at higher concentration due to the near linear decrease of *S*_*a*_ with the increased carrier concentration. Meanwhile, the anisotropy of *S*_*a*_ and *S*_*c*_ is observed with weak dependence on the thickness of SnS_2_ nanosheets, because the Seebeck coefficients along two directions exhibit similar increasing behavior with the layers of nanosheet decreased from 15 L to 3 L. At a given carrier concentration, the increased Seebeck coefficient with the thickness diminishing originates from the enlarged DOS slope of conduction band.

A trade-off should be rendered between Seebeck coefficient and electrical conductivity to obtain high power factor (*PF* = *S* ^2^ *σ*), owing to their opposite response to the carrier concentration. Consequently, the *PF* values of the multilayer SnS_2_ nanosheets as functions of carrier concentration are calculated and plotted in Fig. 3(c). The *PF* along a direction is about 6 orders of magnitude higher than that along *c* direction, suggesting the thermoelectric properties of SnS_2_ nanosheets are more promising along *a-b* plane than *c* direction. The electronic thermal conductivities in the *a* and *c* directions are obtained from the Wiedemann-Franz law, namely, *κ*_*e*_ = *LσT*, where the Lorenz number *L* is approximately 1.5 × 10^−8^ WΩK^−2^. Consequently, the *κ*_*e*_-*n* curves exhibit similar trends to electrical conductivity as shown in Fig. 3(d). The anisotropy of electronic thermal conductivity is pronounced with the thickness of SnS_2_ nanosheets diminishing, which agrees with the trend of *σ* and *PF*.

**Figure 4:**
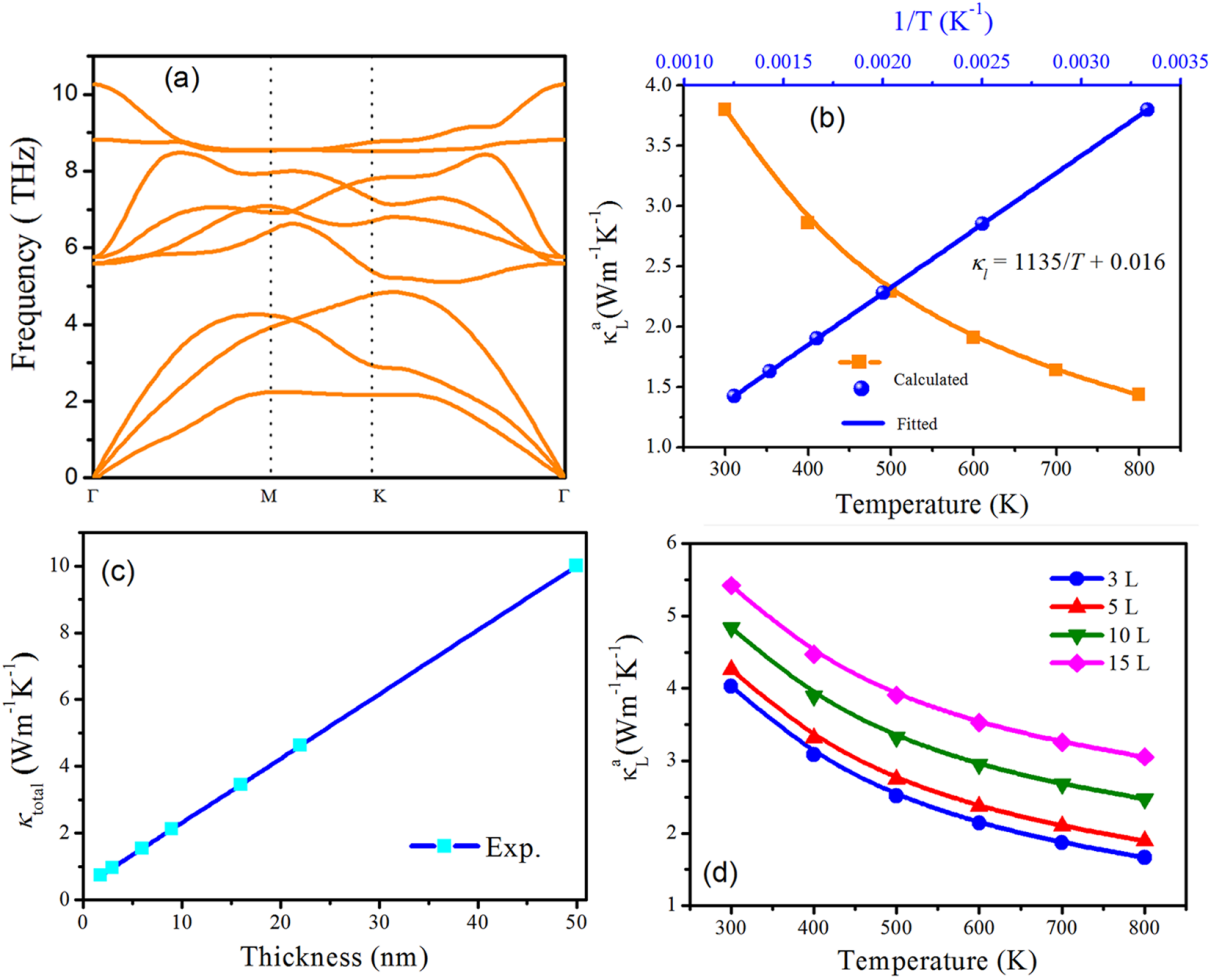
Phonon dispersions along high symmetry of monolayer SnS_2_ (**a**). Calculated lattice thermal conductivity of monolayer SnS_2_ as a function of temperature. (**b**) The fitted lattice thermal conductivity according to experimental reports as a function of thickness (**c**). Simulated lattice thermal conductivity of SnS_2_ nanosheets for various layers as a function of temperature (**d**).

The total thermal conductivity consists of electronic thermal conductivity and lattice thermal conductivity. In the classic phonon transportation theory, the lattice thermal conductivity decrease with an increase of temperature, following the universal 1/*T* relation. The calculated lattice dynamic properties of single-layer SnS_2_ sheet are calculated to estimate the similar nanosheet samples. The phonon dispersion obtained from the force constants is shown in Fig. 4(a). There are no imaginary frequencies in the phonon spectra implying the thermodynamical stability of SnS_2_ nanosheet. There exist 3 acoustic and 6 optical phonon branches corresponding to 3 atoms per unit cell. The three lowest phonon branches are acoustic phonon branches. The in-plane longitudinal acoustic (LA) branch and transversal acoustic (TA) branch have linear dispersions as the wave vector approaches Γ point. The out-of-plane ZA branch is flexural due to the rapid decay of transversal forces, which is similar to other 2D materials^43–45^. Based on the harmonic and anharmonic IFCs, the lattice thermal conductivity (*κ*_L_) is calculated by solving the linearized BTE for phonons. By employing the iterative method, the obtained *κ*_L_ of single-layer SnS_2_ nanosheet at different temperatures are listed in Fig. 4(b). The lattice thermal conductivity decreases with the temperature elevating. The fitted line well satisfies the 1/*T* relationship, indicating that anharmonic phonon–phonon interactions are dominant in the phonon scattering mechanism. It should be mentioned that the calculated thermal conductivity of the single-layered SnS_2_ sheet is larger than the total thermal conductivity obtained from the fitted values according to the experiment results of thin films^30^. The fitted line of thickness (*t*) dependent *κ* well satisfies the relationship $\,\kappa \propto \,\frac{1}{t}$as plotted in Fig. 4(c). The strong phonon scatterings at internal boundaries and the surface induced by dimension decreasing give rise to the reduction of *κ*. Assuming that the thickness dependence of calculated thermal conductivity of SnS_2_ multilayers adheres to the same $\frac{1}{t}$ relationship, we can obtained theoretical lattice thermal conductivity of SnS_2_ nanosheets with different layers at room temperature. Furthermore, the lattice thermal conductivity of multilayer SnS_2_ nanosheets at temperature from 300 K to 800 K can be yield according to the 1/T relation of single-layer sheet (see Fig. 4d).

The calculated lattice thermal conductivity is much higher than the fitted total thermal conductivity from the experimental values, which originates from the following reason: in first principles approach only the phonon-phonon scattering is considered while in samples used for experimental measurement contain other types of scattering due to defects, impurities and dislocations which in turn reduce the thermal conductivity. A similar phenomenon has been reported in many 2D compounds, such as SnSe and phosphorene^45, 46^. The accumulative lattice thermal conductivity of SnS_2_ sheet as a function of MPF at different temperature is shown in Fig. S4 of SI.

**Figure 5:**
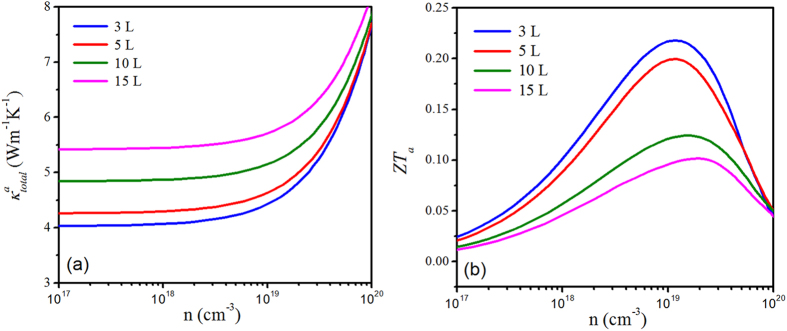
The total thermal conductivity (**a**) and *ZT* (**b**) value in *a-b* plane as a function of carrier concentration at room temperature.

The total thermal conductivity of multilayer SnS_2_ nanosheets in *a-b* plane as a function of carrier concentration is shown in Fig. 5(a). At mid-low carrier concentration, the lattice part makes major contribution to the total thermal conductivity. With all the available transport coefficients, the *ZT* values of SnS_2_ nanosheets with different layers at room temperature as a function of carrier concentration are shown in Fig. 5(b). It can be found that the *ZT* peak values increase with the numbers of layers decreasing. The 3 L SnS_2_ nanosheets yield *ZT* maximum of 0.22 along *a-b* plane at the optimal carrier concentration 1.4 × 10^19^ cm^−3^ at room temperature. The obtained maximal *ZT* value of 2D SnS_2_ nanosheets is slight higher than the calculated optimal value of bulk SnS_2_ (0.19) at 300 K due to the overestimated thermal conductivity.

**Figure 6:**
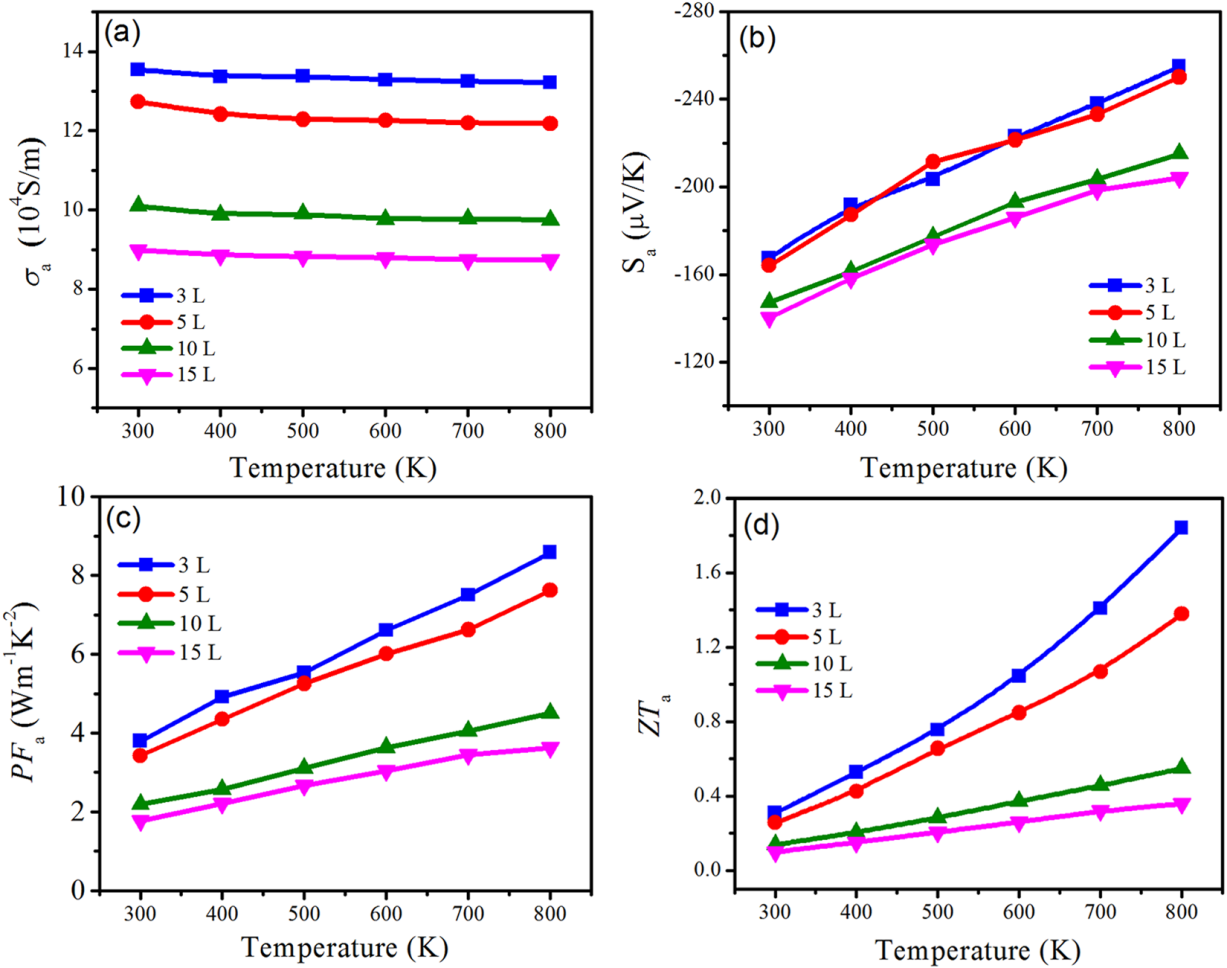
Electrical conductivity (**a**), Seebeck coefficient (**b**), power factor (**c**), *ZT* (**d**) of SnS_2_ sheets along *a-b* plane for 3 L, 5 L, 10 L, 15 L as functions of evaluated temperature at optimal carrier concentration.

To examine the effect of temperature on the thermoelectric performance of multilayer SnS_2_ nanosheets, the transport parameters *σ*_*a*_, *S*_*a*_, *PF*_*a*_ and *ZT*_*a*_ value as functions of temperature are systematically calculated. The values at optimal carrier concentration (~1.45 × 10^19^ cm^‒3^) of SnS_2_ sheets in different layers are extracted and plotted in Fig. 6(a–d). As shown in Fig. 6(a), the electrical conductivity of samples in various layers shows similar temperature independence from 300 K to 800 K. In contrast to *σ*_*a*_, the *S*_*a*_ shows increasing trend with temperature and the 3 L and 5 L SnS_2_ sheets exhibit higher *S* value. The power factor of multilayer sheets in *a-b* plane at optimal doping concentration as a function of temperature can be available in Fig. 6(c). It can be found the *PF*_*a*_ increases with the evaluated temperature. Compromising the negative relevance of *σ* and *S* to temperature and carrier concentration, the SnS_2_ nanosheets of 3 L achieves the maximum *σS* ^2^ value. At the optimal carrier concentration, neither the lattice thermal conductivity nor the electronic thermal conductivity can be negligible. Consequently, we obtained the maximum *ZT* values at the optimal doping concentration at different temperature, as shown in Fig. 6(d). At 800 K, the *ZT* value of 3 L SnS_2_ nanosheets reaches the maximum of 1.87 at optimal carrier concentration. Although the thermal conductivity is over estimated, the calculated *ZT* value of 2D SnS_2_ sheets in *a-b* direction is significantly higher than that of bulk SnS_2_, satisfying the commercial demands and indicating great potential as a 2D high performance thermoelectric material.

## Conclusion

In summary, by combining the DFT first principle calculations and the semi-classical Boltzmann transport theory, we systematically investigate the thickness dependence of electronic and thermoelectric transport properties of ultrathin SnS_2_ nanosheets in various layers. With the thickness decreasing from about 9 nm (15 L) to about 1.8 nm (3 L), the electrical conductivity and Seebeck coefficient increased simultaneously. And the enhancement becomes pronounced for the 5 L sample (around 3 nm), which is approaching the exciton Bohr radius (3.64 nm) of bulk SnS_2_. We demonstrate that the enhanced electron density interlayers, originating from the electron accumulation due to the dimension confinement effect, accounts for the improvement of *σ*. The increased slope of density of states of conduction band near the Fermi level contributes to the enhancement of *S*. Furthermore, the increased phonon scattering resulting from the strong boundary effect decreases the thermal conductivity. Therefore, the negative response of power factor and thermal conductivity to the thickness variation of SnS_2_ nanosheets contributes to the excellent *ZT* value of 1.87 for 3 L sample in *a-b* plane with appropriate concentration at 800 K. The results reveal the excellent thermoelectric behavior of ultrathin SnS_2_ nanosheets, shedding light on searching promising two dimensional high performance thermoelectric materials.

## Methods

The structure optimization and electronic structure calculations for multilayers SnS_2_ nanosheets are done within density functional theory (DFT) and plane wave pseudopotential technique, as implemented in the *Vienna Ab-initio Simulation Package* (VASP)^47^. The vacuum layer size, set as 18 Å along the crystallographic *c*-axis ensures that interactions between the layers are negligible. The generalized gradient approximation of Perdew-Burke-Ernzerhof (PBE)^48^ for the exchange-correlation potential and the projector augmented wave (PAW) method^49^ are employed in this code. The Van der Waals interaction is considered by adding a semi-empirical dispersion potential to the conventional Kohn–Sham DFT energy, through a pair-wise force field following Grimme’s DFT-D2 method^32^. The kinetic energy cutoff of wave functions is 500 eV, with the energy convergence sets as 10^−4^ eV/atom. A Monkhorst-Pack *k*-point mesh of 15 × 15 × 1 used to sample the Brillouin zones in structural optimization and self-consistent calculation. As exact electrical calculation is important for the accurate prediction of thermoelectric transport properties, the denser *k*-point mesh convergence test is performed and consequently 35 × 35 × 1 is employed for electronic structure. We use the semiclassical Boltzmann transport theory implemented in BoltzTrap code^39^ to calculate the doping concentration dependence of transport coefficients. The constant relaxation time approximation and rigid band model were used in this package. The harmonic interatomic force constants (ICFs) using 5 × 5 × 1 supercells calculated by Phonopy package^50^, and anharmonic ICFs using 5 × 5 × 1 supercells created by ShengBTE code^51^. The lattice thermal conductivity is calculated by solving the linearized BTE for phonons. The VESTA software^52^ was used for visualization and the charge density plotting.

## Electronic supplementary material

Supplementary Information
