# Supplementary material for: Thickness-controlled electronic structure and thermoelectric performance of ultrathin SnS2 nanosheets
Source: Sci Rep. 2017 Aug 21;7:8914. doi: 10.1038/s41598-017-09572-9 (PMC5567102; doi:10.1038/s41598-017-09572-9)
Supplement: Supplementary file 1 — Supplementary Information [file 41598_2017_9572_MOESM1_ESM.pdf]

Supplementary Information

Thickness-controlled electronic structure and thermoelectric performance of ultrathin SnS_2 nanosheets

Jun Li[1,2], Jinni Shen[3], Zuju Ma[1], & Kechen Wu[1,*]

[1]*State Key Laboratory of Structural Chemistry, Fujian Institute of Research on the Structure of Matter, Chinese Academy of Sciences, Fuzhou, Fujian 350002, People's Republic of China.*

[2]*University of Chinese Academy of Sciences, Beijing 100049, People's Republic of China.*

[3]*College of Materials Science and Enginerring, Fuzhou University, Fuzhou350108, People's Republic of China.*

*Correspondence and requests for materials should be addressed to K.C.W. (e-mail: wkc@fjirsm.ac.cn)

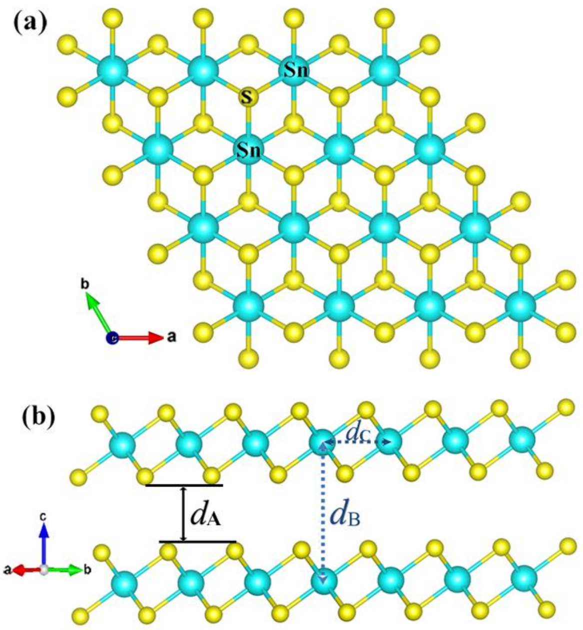

Figure S1. (a) A top view of SnS_2 nanosheets. (b) Side view. Here, blue and yellow colors represent Sn and S atoms, respectively. The vertical distance between layers is defined as d_A, the chalcogen distance of interlayer and *a*-*b* plane are defined as d_B and d_C, respectively.

Table S1. Geometric parameters of SnS_2 nanosheets in various layers. The unit of bond length and interatomic distance is in Å.

Layers	Bond length	d_A	d_B	d_C	∠S-Sn-S
3	2.583	3.105	5.792	3.6433	90.015
5	2.581	2.975	5.894	3.6430	90.019
10	2.578	2.918	6.090	3.6428	90.156
15	2.577	2.892	6.212	3.6427	90.236

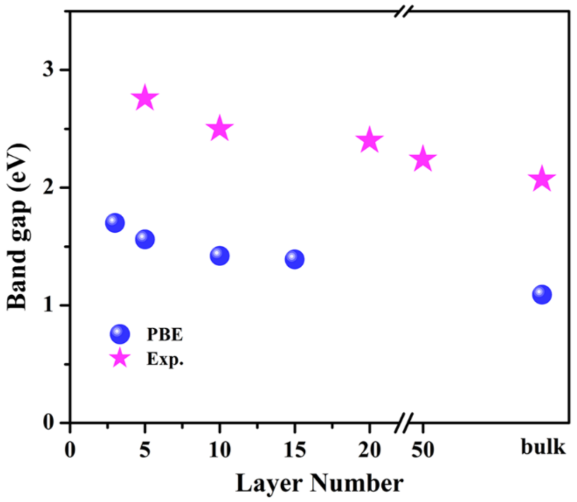

Figure S2. The calculated (ball in blue) and experimental [a] (star in pink) band gap of SnS_2 from bulk [b] to 2D sheet of various layers.

a. Reference [1]

b. Reference [2]

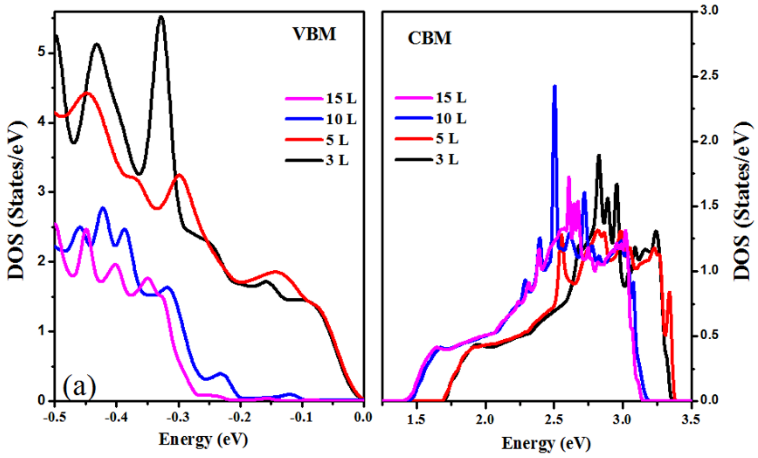

Figure S3. The density of states (DOS) of SnS2 nanosheets in various layers for the (a) VBM and (b) CBM. The Fermi energy level is shifted to 0 eV.

The cumulative thermal conductivity as a function of the phonon mean free path (MPF) at temperature from 300 K to 800 K is fitted to a single parametric function

$$\kappa\ (l \leq l_{max}) = \kappa_0/(1+l_0/l_{max})$$

κ_0 is the ultimate cumulated κ, l_{max} is the maximal MPF concerned, and l_0 is the parameter to be evaluated by the fitting. The fitted curves reproduce the calculated data quite well and yield the parameter l_0, which would be interpreted as the representative MPF. The cumulative thermal conductivity with respect to MPF is helpful to predict the size effect on the phonon transport.

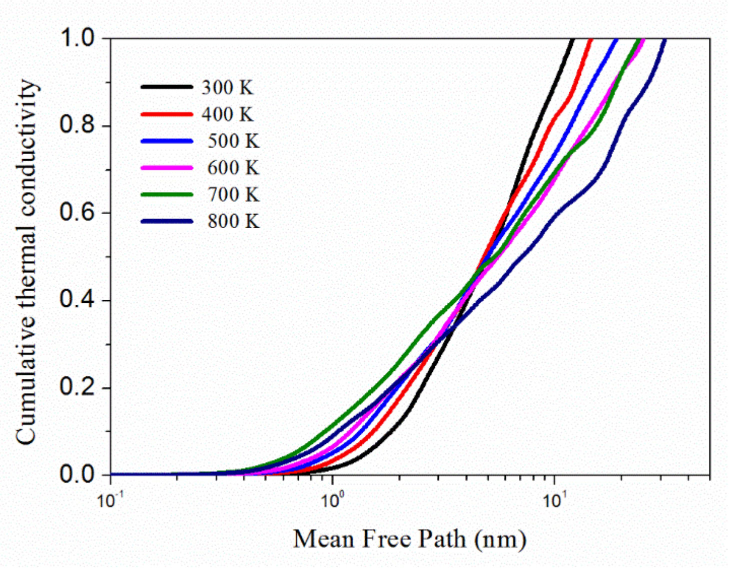

Figure S4. The accumulative lattice thermal conductivity of single-layer SnS_2 sheet as a function of MPF at different temperature.

1. Lee, M.-J. *et al*. Thermoelectric materials by using two-dimensional materials with negative correlation between electrical and thermal conductivity. *Nat. Commun.* **7**, 12011 (2016).
2. Sun, B.-Z., Ma, Z.-J., He, C. & Wu, K.-C. Anisotropic thermoelectric properties of layered compounds in SnX_2 (X=S, Se): a promising thermoelectric material. *Phys. Chem. Chem. Phys.* **17**, 29844–29853 (2015).
